# Multiomics investigation of the female hypertensive human heart

**DOI:** 10.14814/phy2.70586

**Published:** 2025-10-02

**Authors:** Zachary J. Milstone, Jesse D. Moreira‐Bouchard, Karan K. Smith, Anjali Anilkumar, Adam C. Gower, Yuriy Alekseyev, Jason A. Cunha, Nathaniel Fisher, Levi Legler, Joshua Lepson, Brian S. Tao, Christopher Williams, Emelia J. Benjamin, Daniel Levy, Richard N. Mitchell, Robert F. Padera, Hardik Shah, Seung Hoan Choi, Deepa M. Gopal, Nathan R. Tucker, Jessica L. Fetterman

**Affiliations:** ^1^ Evans Department of Medicine and The Whitaker Cardiovascular Institute Boston University Chobanian & Avedisian School of Medicine Boston Massachusetts USA; ^2^ Department of Health Sciences, Programs in Human Physiology, Sargent College of Health & Rehabilitation Sciences Boston University Boston Massachusetts USA; ^3^ Department of Medicine, Section of Computational Biomedicine, and Clinical and Translational Science Institute Boston University Chobanian & Avedisian School of Medicine Boston Massachusetts USA; ^4^ Department of Pathology and Laboratory Medicine Boston University Chobanian & Avedisian School of Medicine Boston Massachusetts USA; ^5^ Department of Pharmacology and Medicine SUNY Upstate Medical University Syracuse New York USA; ^6^ Section of Cardiovascular Medicine, Department of Medicine, Boston Medical Center Boston University Chobanian & Avedisian School of Medicine Boston Massachusetts USA; ^7^ Department of Epidemiology Boston University School of Public Health Boston Massachusetts USA; ^8^ Framingham Heart Study Framingham Massachusetts USA; ^9^ Population Sciences Branch National Heart, Lung, and Blood National Institutes of Health Bethesda Maryland USA; ^10^ Department of Pathology Brigham and Women's Hospital, Harvard Medical School Boston Massachusetts USA; ^11^ University of Chicago Medicine Comprehensive Cancer Center Metabolomics Platform Chicago Illinois USA; ^12^ Department of Biostatistics Boston University School of Public Health Boston Massachusetts USA; ^13^ Cardiovascular Disease Initiative Broad Institute of MIT and Harvard Cambridge Massachusetts USA

**Keywords:** biobanking, cardiac metabolism, hypertension, multiomics

## Abstract

Hypertension affects 1 in 2 adults in the United States and is the leading risk factor for myocardial infarction and chronic kidney disease. While animal models have advanced our understanding of the effects of hypertension on the heart, molecular insight from human cardiovascular tissues is currently lacking. Building upon previous work describing a protocol for the systematic dissection and preservation of whole postmortem human hearts, we performed pilot multiomics analyses of postmortem human hearts from donors with (*n* = 3) and without (*n* = 2) hypertension. Using bulk RNA‐seq, we identified a higher abundance of transcripts associated with DNA helicase activity, NAD‐dependent deacetylase activity, and branched chain amino acid metabolism in hypertension compared to normotension. Using single‐nucleus RNA‐seq, we identified a loss of contractile vascular smooth muscle cells and greater endothelial cell proliferation associated with hypertension. Lastly, metabolomics revealed an abundance of metabolites upstream of NAD‐dependent metabolic steps in fatty acid oxidation and the Krebs cycle, consistent with reductive stress, and a likely funneling of glycolytic intermediates into the pentose phosphate pathway. Together, these methods demonstrate a powerful technique for the investigation of human cardiovascular disease and lend insight into the molecular signature of hypertension in adult cardiac tissue.

## 1 INTRODUCTION

Hypertension affects ~50% of US adults, is a leading heart failure risk factor, and contributes $80 billion to US health expenditures annually (Martin et al., 2024). Only 1 in 5 patients with hypertension achieves blood pressure control, partially attributable to the nontargeted nature of current antihypertensives, underscored by our lack of mechanistic insight into hypertensive molecular mechanisms. As such, there is a need for improved therapies (World Health Organization, 2025). Animal models are useful for mechanistic evaluation of individual cardiovascular disease risk factors but cannot fully recapitulate the complex physiology of hypertensive hearts, leading to the need to develop innovative approaches to study human cardiac biology in hypertension.

Clinically, direct access to cardiac tissues is restricted to left atrial appendage and right ventricular biopsies. Consequently, investigations into the chamber‐specific biology of the hypertensive human heart have not previously been done. Biobanked, postmortem human heart tissue presents a unique opportunity to understand the pathogenesis of human cardiovascular diseases through detailed multiomics analyses. Here, we examine the effects of hypertension on the cardiac transcriptome, cellular subtypes, and metabolic profile. We performed bulk RNA sequencing (RNA‐seq), single‐nucleus RNA‐seq (snRNA‐seq), and metabolomics on ventricular and atrial samples from donors with hypertension (*n* = 3) and normotensive referents (*n* = 2). With these analyses, we identified cardiac site‐specific cellular and molecular signatures associated with hypertension.

## 2 METHODS

### 2.1 Heart procurement, dissection, and preservation

Postmortem human hearts were obtained from the National Disease Research Interchange. No contact was made by the investigators with human participants and deidentified donor information was provided to the investigators. The Boston University Institutional Review Board deemed Protocol H‐42979 exempt. Hearts were stored during transport in cardioplegic solution (Belzer UW Cold Storage Solution: 320 mOsm, [Na+] = 29 mEq/L, [K+] = 125 mEq/L, pH = 7.4 at 20°C), systematically dissected, flash frozen, and stored to maintain the anatomical registration for multiomics as previously described (Moreira et al., 2023).

### 2.2 Multiomics

#### 2.2.1 Bulk RNA‐seq

RNA was extracted from right (RV) and left (LV) mid‐ventricular wall samples using the Qiagen RNeasy Fibrous Tissue Mini Kit. RNA recovery was quantified using a NanoDrop N‐D 1000 spectrophotometer. Libraries were prepared with ribosomal RNA depletion utilizing the Kapa Roche KK8561KAPA RNA Hyper+RiboErase HMR and sequenced on an Illumina NextSeq 2000 instrument by the Boston University Microarray and Sequencing Resource core. For bulk RNA‐seq data, reads were mapped with STAR and read counts were analyzed across samples by Wald tests using *DESeq2* to determine differential RNA expression, with age used as a covariate, as previously described (Moreira et al., 2023). Principal component analysis (PCA) was performed using differentially expressed genes (FDR < 0.05). Gene Set Enrichment Analysis was performed using gene sets from the Molecular Signatures Database (MSigDB) and plotted with variance stabilizing transformed expression values for each gene, adjusted for site and then *z*‐score‐normalized to a mean of zero and standard deviation of 1 across all samples in each row indicating final *z*‐scores of ≤ −2, 0, and ≥ 2, respectively.

#### 2.2.2 snRNA‐seq

Single nuclei suspensions were isolated from flash frozen tissue samples and processed for single nucleus RNA sequencing using 10× Genomics Chromium Next GEM Single Cell 3′ v3.1 as previously described (Tucker et al., 2020) with an estimated 5000 nuclei per device recovered. Fastqs were processed to a count matrix using CellRanger v7.0.1. Ambient RNA was removed and droplets containing valid nuclei were called using CellBender v0.2 (Fleming et al., 2023). The resultant gene count matrix was processed using Scanpy (Satija et al., 2015; Wolf et al., 2018). The dataset, which included 10 samples consisting of five pairs of atrial and ventricular tissues, underwent quality control to remove low‐quality cells and genes. Metrics necessitating removal included: cells containing less than 200 genes, genes that are present in less than 3 cells, and cells containing a mitochondrial gene content greater than 5%. Doublet scores were calculated using Scrublet (Wolock et al., 2019). Cells with doublet scores above 0.12 were removed.

For clustering, the top 500 highly variable genes were identified using Pearson residuals (Lause et al., 2021). PCA was performed using those genes followed by integration of samples using Harmony (Korsunsky et al., 2019). Following this, Harmony‐adjusted PCs were used for nearest neighbor calculation and Leiden clustering. Cell type assignment was conducted at the cluster level using a panel of canonical marker genes and known cardiac resident cell labels, including *SORBS2* (cardiomyocyte), *MYH7* (cardiomyocyte), *PECAM1* (endothelial), *VWF* (endothelial), *DCN* (fibroblast), *MYH11* (vascular smooth muscle), *PDGFRB* (pericyte), *CD163* (macrophage), *MRC1* (macrophage), *SKAP1* (lymphocytes), *PTPRC* (lymphocytes), *NRXN1* (neuronal), and *NCAM2* (neuronal). Vascular smooth muscle cells (VSMCs) were identified by abundant MYH11 expression.

To resolve subtypes, we performed reciprocal nonparametric differential expression testing (Wilcoxon rank‐sum) between communities identified by Leiden clustering at a resolution of 0.15. Genes upregulated in each contrast (*p* < 0.05) were subjected to Gene Ontology enrichment analysis, and subtype labels were assigned using the enriched biological processes identified from the corresponding gene set. Contractile VSMCs were identified based on upregulation of genes associated with smooth muscle contraction. Mesenchymal‐like VSMCs were identified based on the enrichment of genes associated with the regulation of the transition from an epithelial cell state to a mesenchymal cell state.

The dataset was subsequently split into atrial and ventricular subsets for chamber‐specific analysis or into cell type specific objects for subclustering analysis. In both instances, the same normalization, clustering, and cell type assignment strategy was applied to each subset independently. Wilcoxon rank sum test was used to calculate marker gene cell type specificity with a *p* value cutoff of 0.0001. For all comparative analysis between clusters through one‐versus‐all approaches, cell down sampling was applied during statistical testing to match the cell count of the smallest cluster to reduce the impact of more numerous populations. To facilitate improved clustering and as a form of more stringent ambient RNA correction, genes specific to cardiomyocytes with a corresponding *p* value less than 0.0001 were removed from non‐cardiomyocyte cell types (6300 genes). Ranked genes with a *p* value less than 0.05 were used in a hypergeometric test for gene ontology (GO) enrichment analysis (Ashburner et al., 2000; Gene Ontology Consortium et al., 2023; Thomas et al., 2022).

For analysis of cell cycle, counts were obtained at the cell type level for each identified subcluster and used for comparative analysis of population changes across hypertensive and nonhypertensive condition states. S phase and G2M phase cell cycle scores were calculated using the “Score cell cycle genes” tool in Scanpy. Composite cell cycle scores were calculated by combining the S and G2M scores. Significant alterations in cell cycle parameters were determined using the Kolmogorov–Smirnov test and the Mann–Whitney *U* test.

An annotated data object containing PCA scores and a UMAP embedding generated using the previously described Scanpy pipeline, was round‐tripped to R as a SingleCellExperiment using zellkonverter::readH5AD. Scanpy embeddings were mapped to reducedDim(sce, “PCA”) and reducedDim(sce, “UMAP”). Neighborhoods were defined with miloR on the PCA space by constructing a k‐nearest‐neighbor graph (*k* = 25, dimensionality set to the number of available PCs), generating refined neighborhoods, and computing neighborhood–neighborhood distances on the same PCA space. Cells were counted per neighborhood × sample, using the per‐cell sample identifier (“batches”) carried in colData. Differential abundance (DA) testing was performed with miloR's edgeR‐based negative‐binomial GLM using an additive design that adjusted for chamber (~ 1 + chamber + condition); effect sizes are reported as log_2_ fold‐changes in neighborhood abundance between the HTN and NTN conditions. *p* Values were adjusted with miloR's spatial FDR using k‐distance weighting, and significance was defined a priori (SpatialFDR < 0.15). Neighborhoods were annotated to majority labels by aggregating cell‐level metadata (cell type, condition, and chamber), and summary tables (counts of significant neighborhoods by cell type and condition) were generated from the DA results. For visualization, neighborhood centroids were projected onto the UMAP and colored based on DA metrics.

Quality control metrics and metadata can be found in Table S1.

#### 2.2.3 Metabolomics

Untargeted metabolomics was conducted on left and right ventricular (LV and RV) tissues. Polar metabolite profiling was performed similar to the procedure described previously (Yang et al., 2024). In brief, snap‐frozen heart tissue was pulverized into a powder using a mortar and pestle on dry ice. The metabolites were extracted using the ice‐cold 4/4/2 acetonitrile/methanol/water (50 μL solvent per mg of tissue, LC–MS grade solvents), and homogenized (Omni International, TH115‐PCR5H, stainless steel probe). Samples were then vortexed and subjected 2 times to sonication for 4 min in an ice‐cold water bath, frozen in liquid nitrogen for 1 min, thawed on ice and subsequently vortexed for 5 min at 2000 rpm and 4°C using a Thermomixer. Samples were incubated on ice for 20 min, centrifuged at 20,000 × *g* for 20 min at 4°C, and 150 μL of supernatant from each sample was dried down using the Genevac EZ‐2.4 elite evaporator. The dried‐down samples were stored at −80°C until analysis.

On the day of the analysis, the samples were resuspended in 50 μL of 60/40 acetonitrile/water. The LC–MS was performed at the University of Chicago Medicine Comprehensive Cancer Center Metabolomics Platform, as described (Yang et al., 2024). Metabolite abundance data were first column‐sum normalized and log_2_ transformed to improve normality. PCA was performed on normalized data using the prcomp() function from the stats package in R (v.4.4.0) (R Core Team, 2021). We first conducted association testing between postmortem interval (PMI) and the first 10 PCs using generalized linear models (glm() function, *stats* package, R):

$$\mathrm{PMI}\;\left(\text{Hours}\right)\sim \text{PC1}+\text{PC2}+\ldots +\mathrm{PC}10$$

For PCs associated with PMI (*q* < 0.05), metabolites contributing the most to the PC were identified based on their eigenvector loadings. Metabolites with |eigenvector| > 0.15 were removed, while those below this threshold were retained for further analysis. Differential abundance analysis was performed using the Limma R package (v.3.60.4) (Ritchie et al., 2015), which applies linear models with an empirical Bayes method for variance shrinkage. Mean metabolite abundances in LV and RV tissues from each donor were compared between hypertensive and normotensive groups. Additionally, metabolite abundance was analyzed with and without samples from donors with type 2 diabetes in a sensitivity analysis.

#### 2.2.4 Bioinformatics and statistical analysis

For analysis, we opted to group samples according to disease state (HTN vs. NTN) and use high‐level pathway enrichment analyses rather than blood pressure as a continuous variable to avoid overfitting our small sample size. As this was a pilot study, we focus on high order biology rather than individual gene or metabolite differential expression. For all statistical testing, a significance threshold of an FDR *q* < 0.05 was used unless otherwise noted.

## 3 RESULTS

### 3.1 Donor characteristics

**FIGURE 1:**
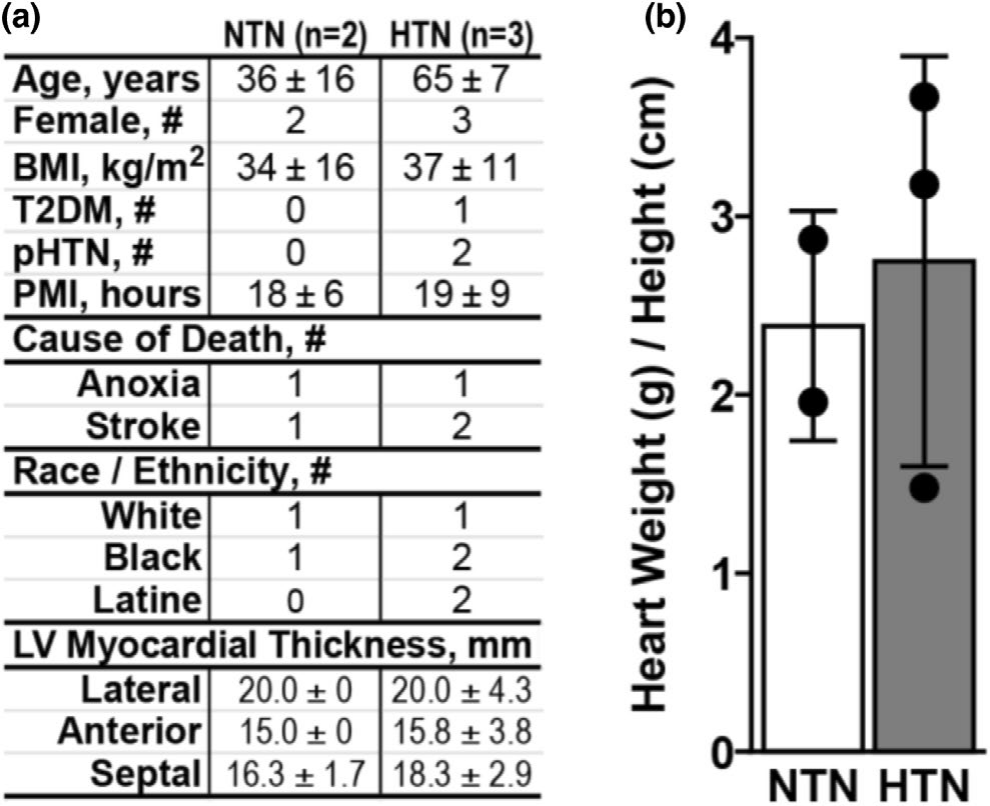
Donor demographics and heart characteristics. (a) Demographics of heart donors with (*n* = 3, HTN) and without (*n* = 2, NTN) hypertension and left ventricular (LV) myocardial thickness. BMI, body mass index; pHTN, pulmonary hypertension; PMI, postmortem interval; T2DM, type 2 diabetes mellitus. Data presented +/− standard deviation. (b) Bar graph showing hypertensive and normotensive donor heart weight (g) indexed to donor height (cm). Data presented +/− standard deviation.

All heart donors were female with similar body mass indexes, causes of death, and postmortem intervals (Figure 1a). Hypertensive donors were older (65 ± 7 years) than normotensive donors (36 ± 16 years). Myocardial wall thicknesses (Figure 1a) and index myocardial masses were similar between hypertensive and normotensive donors (Figure 1b), suggesting hypertensive donor hearts had not undergone gross anatomic remodeling.

### 3.2 Bulk RNA‐seq

**FIGURE 2:**
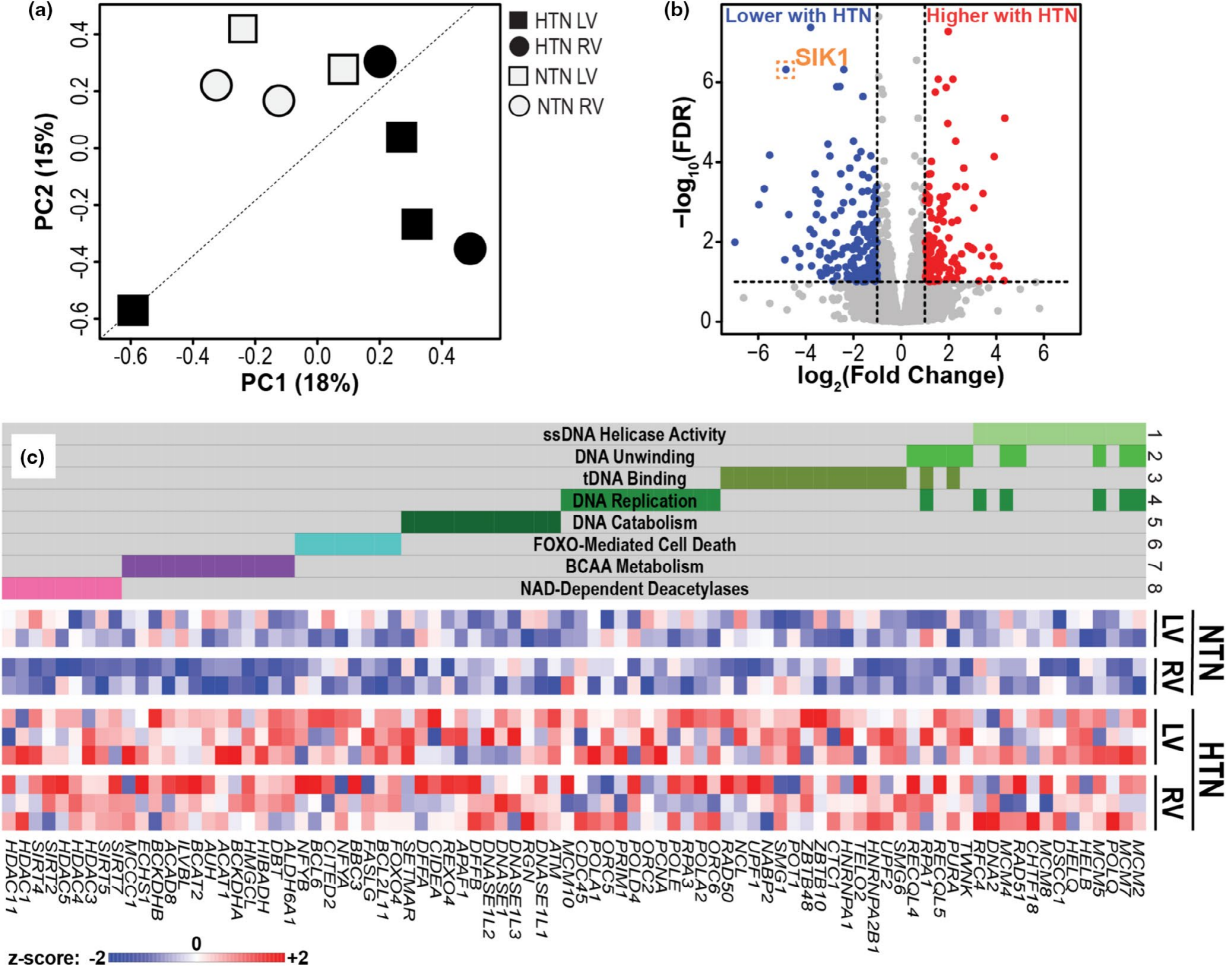
Bulk RNA sequencing of hypertensive versus normotensive left (LV) and right (RV) ventriclar samples demonstrate altered metabolic and gene‐regulatory programs. (a) Principal Component Analysis (PCA) of bulk RNA‐seq expression data, in which samples separate by disease status but not by anatomic site along the diagonal of principal components (PC) 1 and 2. (b) Volcano plot computed from a model of bulk RNA‐seq expression data as a function of HTN after correcting for site (LV or RV). Points in red or blue are significantly (FDR *q* < 0.05) and strongly up‐ (*n* = 97) or down‐ (*n* = 124) regulated (|fold change| > 2), respectively, in hypertension (HTN) versus normotension (NTN). *SIK1*, salt‐inducible kinase 1. (C) Heatmap of the union set of leading‐edge genes from eight of the most significantly coordinately upregulated pathways (FDR *q* < 0.05) as determined by Gene Set Enrichment Analysis, ranking genes by Wald statistics computed for hypertension versus normotension after correcting for site (RV or LV) and using gene sets from the Molecular Signatures Database (MSigDB). Membership of each gene within each gene set is indicated with a colored box above the heatmap. (1) ssDNA helicase activity (GO:0017116); (2) DNA unwinding (GO:0006268); (3) telomeric (t) DNA binding (GO:0042162); (4) DNA replication (WP466); (5) DNA catabolism (GO:0009081); (6) FOXO‐mediated cell death (R‐HAS‐9614657); (7) BCAA metabolism (GO:0009081); (8) NAD‐dependent deacetylases (GO:0034979).

We sought to identify molecular alterations associated with hypertension in human cardiac samples using a multiomics approach. We first performed bulk RNA‐seq on LV and RV tissues from each donor, which segregated by disease state in PCA of gene expression but not by donor site (Figure 2a). There were 230 differentially expressed genes that were strongly and significantly up‐ or down‐regulated in hypertension (FDR *q* < 0.05, |fold change| > 2) (Figure 2b) including *SIK1*, (salt‐inducible kinase 1), which is involved in various processes like cell cycle regulation, metabolism, and tumor suppression (highlighted in orange).

We then performed Gene Set Enrichment Analysis, which revealed coordinately higher upregulation of genes in hypertensive myocardium compared to tissue from normotensive referents that are involved in DNA unwinding and replication, branched‐chain amino acid (BCAA) metabolism, and NAD‐dependent deacetylases (Figure 2c).

### 3.3 snRNA‐seq

**FIGURE 3:**
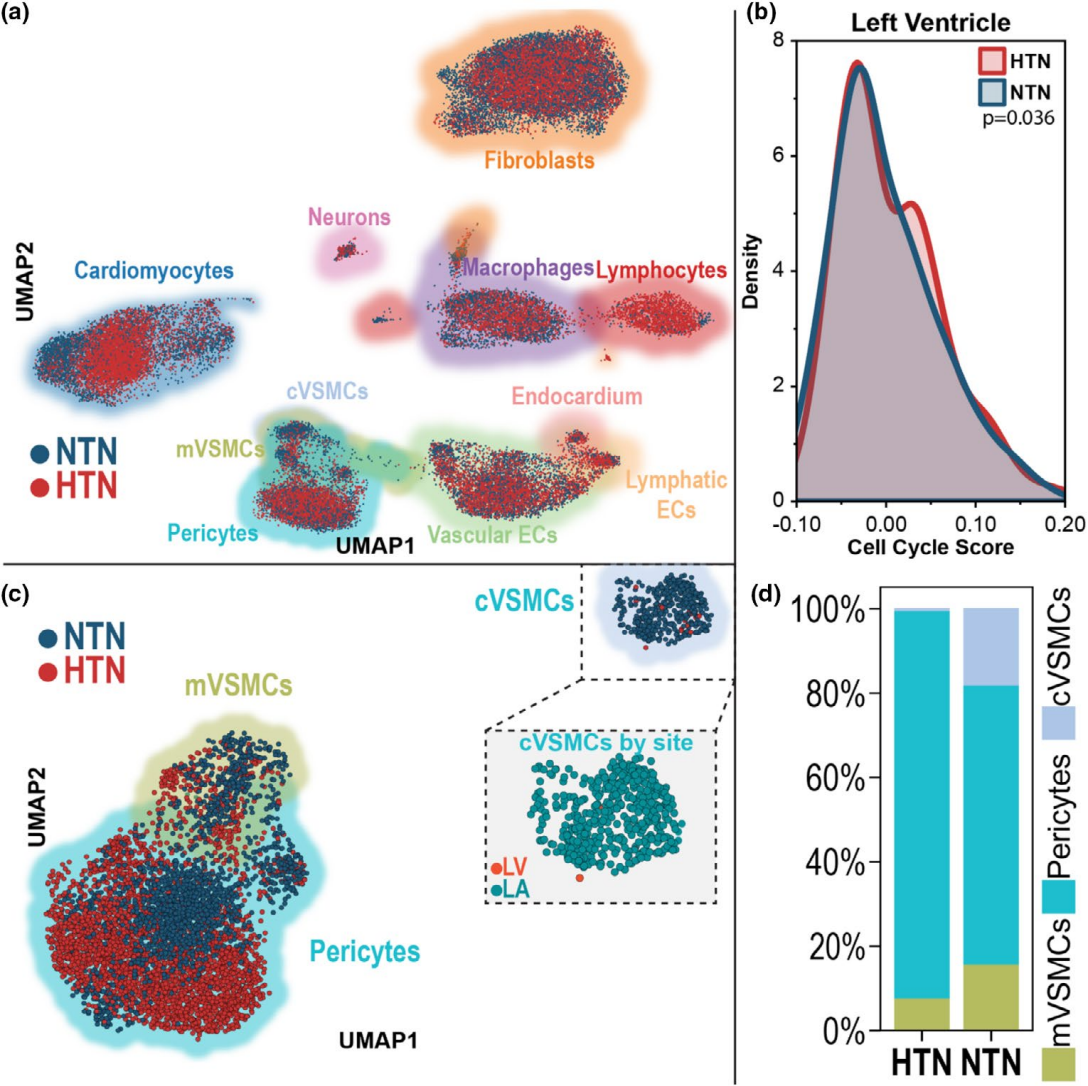
Single‐nucleus RNA sequencing of hypertensive versus normotensive left ventricles and left atria (LA) reveals altered endothelial proliferation and mural cell proportions. (a) Uniform Manifold Approximation and Projection (UMAP) of LV and LA snRNA‐seq samples, grouped by Leiden clustering, colored by disease state, and shaded by cellular subtypes (determined by Boolean putative marker expression). (b) Density plot showing composite cell cycle phase score of LV endothelial cells (ECs), colored by disease state. Significance determined by Mann–Whitney *U* test. (c) UMAP of mural cells colored by disease state and shaded by cell subtype: Contractile vascular smooth muscle cells (cVSMCs), mesenchymal‐like vascular smooth muscle cells (mVSMCs), and pericytes. Inset shows cVSMCs colored by donor site. (d) Stacked bar graph of the relative cell type proportion of combined LV/LA mural cells, defined by snRNA‐seq. FDR *q* < 0.05 for all analyses unless otherwise noted.

To determine the effect of hypertension on myocardial cell type composition and cellular states, we performed snRNA‐seq on a total of 39,201 nuclei isolated from hypertensive and normotensive LV and LA tissues. UMAP dimension reduction revealed cell‐type and disease‐state‐specific diversity (Figure 3a). Amongst these, we isolated a proliferative signal to LV endothelial cells, evidenced by higher composite cell‐cycle density associated with hypertension (Figure 3b). We observed a uniquely atrial contractile vascular smooth muscle cell (cVSMCs) population, which is nearly completely depleted in cardiac tissue from donors with hypertension compared to tissue from donors with normotension (Figure 3c,d).

MiloR differential abundance testing demonstrated enrichment (FDR < 0.15, *p* value <0.05) of the cVSMC population in the NTN condition, (Neighborhoods = 9) when compared to the HTN condition (Neighborhoods = 0) and controlled for chamber effects. We also observe an enrichment (FDR < 0.15, *p* value < 0.05) of vascular endothelial cells in the HTN condition (Neighborhoods = 9) when compared to the NTN condition (Neighborhoods = 4), which is consistent with our findings related to increased expression of proliferation markers in endothelial cells subject to the HTN condition (Figure S1).

### 3.4 Metabolomics

**FIGURE 4:**
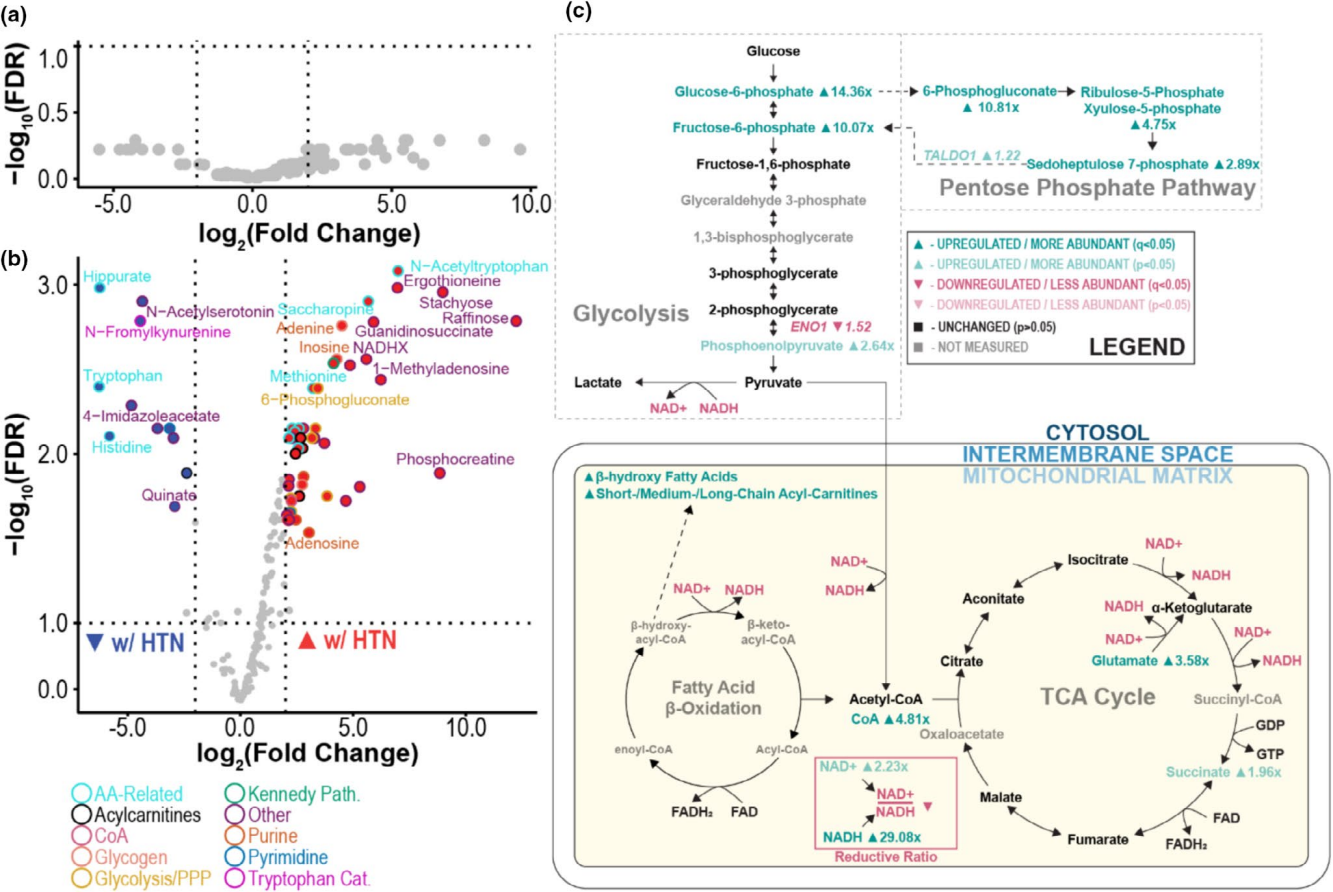
Targeted left ventricular polar metabolite profiling suggests altered NAD^+^/NADH homeostasis in hypertension versus normotension. (a) Volcano plot of LV polar metabolite differential abundance comparing all hypertensive (*n* = 3) versus normotensive samples (*n* = 2) revealed no differentially abundant metabolites (FDR < 0.05). (b) Volcano plot of differential polar metabolite abundance after excluding the hypertensive donor who had type 2 diabetes reveals differential abundance of metabolites associated with amino acid metabolism and glycolysis. (c) Integrated schematic of affected metabolic pathways in hypertension relative to normotension with differentially abundant metabolites and differentially expressed genes at either a significance threshold of FDR *q* < 0.05 (dark) or *p* < 0.05 (light) highlighted (black—not significant, gray—not measured). Fold change and direction of fold change accompanies differentially abundant/expressed metabolites or gene transcripts (green—higher, pink—lower). AA Related, amino‐acid related; CoA, coenzyme‐A; PPP, pentose phosphate pathway; Tryptophan Cat, tryptophan catabolism.

We identified 194 metabolites in total by untargeted metabolomics on left and right ventricular tissue samples. Initial differential abundance analysis revealed no differentially abundant metabolites (Figure 4a, Table S2). One donor with hypertension also had type II diabetes mellitus‐ exclusion of this donor heart (2 hypertensive hearts and 2 referents remaining) allowed for identification of 83 differentially abundant metabolites (*q* < 0.05) between donor hearts with and without hypertension (Figure 4b, Table S2). Several precursors to vasoactive compounds, including tryptophan (nitric oxide precursor, log_2_(FC) = −6.27, *q* = 0.0040) and histidine (histamine precursor, log_2_(FC) = −5.81, *q* = 0.0079), were identified as less abundant in the hypertensive donors, suggesting a potential depletion of vasodilatory agents with important implications for vascular function (Figure 4b).

Several interconnected metabolic pathways had multiple differentially abundant metabolites, such as 6‐phosphogluconate (log_2_(FC) = 3.43, *q* = 0.0041) in the pentose phosphate pathway, glucose‐1‐phosphate (log_2_(FC) = 3.33, *q* = 0.0071) in glycolysis, and glutamate (log_2_(FC) = 1.84, *q* = 0.014), which can feed into the TCA cycle (Figure 4c). Additionally, medium chain fatty‐acyl carnitines and β‐hydroxy fatty acids were more abundant in hypertension compared to normotension, suggesting impaired fatty‐acid oxidation. We identified lower relative amounts of NAD^+^ compared to NADH associated with hypertension compared to normotension, suggesting a shift away from oxidative energy generation, increased reductive stress, and subsequent metabolic pathway feedback inhibition.

## 4 DISCUSSION

We provide evidence of the feasibility of using postmortem human hearts to interrogate disease‐specific molecular and cellular signatures via a multiomics approach. This pilot study revealed several molecular profiles associated with hypertension despite the small sample size and varied donor demographics. Importantly, this study utilized hearts from hypertensive donors that had not undergone gross anatomic remodeling, consistent with an earlier disease process, and provides a unique look into early hypertensive heart disease pathophysiology.

We identified several consistent gene‐regulatory and metabolic signatures associated with hypertension across our multiomic approaches. Collectively, our RNA‐seq and metabolomics suggest altered NAD^+^/NADH homeostasis with concomitant alterations in NAD^+^/NADH‐dependent metabolic and transcriptional processes associated with hypertension. The NAD^+^‐dependent sirtuin deacetylases have demonstrated roles in cardiac hypertrophy and adverse cardiac remodeling in animal models, with activity modulated by NAD^+^/NADH levels, consistent with our metabolomics findings of a lower NAD^+^/NADH in hearts from hypertensive donors (Matsushima & Sadoshima, 2015). Coordinate NAD^+^‐dependent deacetylase upregulation in hypertension suggests a potential role for the sirtuins and reductive stress, defined as disproportionate accumulation of reducing intermediates, including NADH and NADPH, in early hypertensive pathophysiology; however, this difference could be due to the disparate ages of the donors given well‐documented age‐related changes in sirtuin expression levels (Kane & Sinclair, 2018; Kilic et al., 2015).

The altered NAD^+^/NADH ratio also likely contributes to the disruption of multiple NAD‐dependent metabolic reactions, including the accumulation of β‐hydroxy fatty acids and medium‐chain fatty‐acyl carnitines in hypertensive LV tissue. β‐hydroxy fatty acids are the preferred fuel of mature cardiomyocytes, with impaired fatty acid oxidation strongly and consistently associated with cardiovascular disease (Fillmore et al., 2014). Taken with our findings, this suggests altered fatty acid metabolism may contribute to early hypertensive heart disease pathogenesis.

We observed a higher abundance of many metabolites involved in the early steps of glycolysis in our hypertensive cardiac samples, as well as those in the pentose phosphate pathway. Given the increased relative abundance of NADH compared to NAD^+^, we suspect a lower NAD^+^/NADH ratio is exerting feedback inhibition of glycolysis, with resultant alternative pathways activated. Our data points to a funneling of the glycolytic intermediate glucose‐6‐phosphate to 6‐phosphogluconate, an intermediate in the pentose phosphate pathway, suggesting preferential activation of the pentose phosphate pathway. Excess activation of the pentose phosphate pathway has been observed in pulmonary hypertension and contributes to the accumulation of reductive intermediates, including NADPH (Hashimoto & Gupte, 2017).

Additionally, we observed coordinately higher expression of genes associated with BCAA metabolism in hypertensive cardiac tissue. This aligns with previous work showing an association of elevated circulating BCAA levels with hypertension and other cardiometabolic diseases (Fine et al., 2024). Our data suggest that the heart may contribute to higher circulating BCAAs in hypertension, which requires further investigation.

We identified a proliferative transcriptional expression program related to DNA unwinding and replication in both our RV and LV bulk RNA‐seq. snRNA‐seq data allowed us to isolate this proliferative signal to the cardiac endothelial cell population in hypertensive donor hearts. Cardiac endothelial cell proliferation may reflect compensatory angiogenesis driven by the pro‐hypertrophic stimuli of systemic hypertension (Gogiraju et al., 2019). Interestingly, our donors did not have grossly hypertrophied hearts. In the absence of overt hypertrophy, it is possible that coronary vascular dysfunction and altered metabolic programs in the myocardium may relate to angiogenic signaling in advance of gross hypertrophic remodeling, although this remains to be investigated.

Leveraging snRNA‐Seq, we were able to identify site‐specific cell types associated with hypertension. Mural cells, composed of pericytes and vascular smooth muscle cells, are a heterogenous group of resident cardiac cell types that influence a variety of processes associated with hypertension (van Dijk et al., 2015). We found a near‐complete absence of left atrial cVSMCs in cardiac tissue from hypertensive donors. Little is known about the role of this pericyte subtype in the development of hypertensive heart disease; however, a loss of cVSMCs has been demonstrated in animal models of atherosclerosis and is thought to contribute to atherosclerotic pathogenesis (Grootaert & Bennett, 2021). Cardiac atrial‐specific cVSMC loss has not yet been identified as a consequence of hypertension in human tissues and represents a novel finding. Further research is needed to better understand the role of atrial cVSMC depletion in hypertension and the functional consequences of cVSMC loss.

## 5 LIMITATIONS

Although our sample size is limited, the collected cardiac biospecimens represent a rare snapshot into hypertensive heart disease before gross anatomic remodeling. With our small sample size, it is likely that only genes and metabolites that had a large effect size on the difference between disease groups were detected. Future studies, with larger sample sizes, are needed to understand the contributions of altered reductive stress to transcriptional and metabolic alterations associated with hypertension and to determine the contributions of unique VSMC cell states in hypertensive heart disease. The phenotypic data reported with each donor heart was a combination of self‐reported information and limited chart documentation. Important information, such as specific antihypertensive regimens, was not available for some donors. More comprehensive phenotyping and clinical profiles would improve the ability to determine associations between cardiac tissue biology and disease types.

## 6 CONCLUSION

We performed site‐specific multiomics on paired postmortem human hearts and identified both chamber‐ and cell‐type‐specific transcriptomic profiles, a transcriptomic and metabolomic signature of reductive stress, dysregulated BCAA metabolic gene expression, and depletion of atrial cVSMCs associated with hypertension compared to tissue from normotensive referents. Future efforts aimed at pairing detailed spatial multiomics with rich phenotypic data may help to identify novel therapeutic targets in human cardiovascular disease.

## CONFLICT OF INTEREST STATEMENT

The authors declare no conflicts of interest.

## ETHICS STATEMENT

Postmortem human hearts were obtained from the National Disease Research Interchange. No contact was made by the investigators with human participants and deidentified donor information was provided to the investigators. The Boston University Institutional Review Board deemed Protocol H‐42979 exempt.

## Supporting information


Figure S1.

Table S1.

Table S2.

## Data Availability

Count‐level data is available on Gene Expression Omnibus under GEO Series ID GSE282618.
