# Supplementary material for: Multiomics investigation of the female hypertensive human heart
Source: Physiol Rep. 2025 Oct 2;13(19):e70586. doi: 10.14814/phy2.70586 (PMC12489463; doi:10.14814/phy2.70586)
Supplement: Supplementary file 1 — Figure S1. [file PHY2-13-e70586-s001.docx]

**DATA SUPPLEMENT**

**Title**: Multiomics Investigation of the Hypertensive Human Heart

Zachary J. Milstone, MD, PhD^1^*, Jesse D. Moreira-Bouchard, MS, PhD ^1,2^*, Karan K. Smith, BS^1^, Anjali Anilkumar, BA^1^, Adam C. Gower, PhD^3^, Yuriy Alekseyev, PhD^4^, Jason A. Cunha ,MS^1^, Nathaniel Fisher, MS^1^, Levi Legler^5^, Joshua Lepson, BS^1^, Brian S. Tao, BS^1^, Christopher Williams, BS^4^, Emelia J. Benjamin, MD, ScM^6,7^, Daniel Levy, MD^8,9^, Richard N. Mitchell, MD, PhD^10^, Robert F. Padera, MD, PhD^10^, Hardik Shah, MS^11^, Seung Hoan Choi, PhD^12^, Deepa M. Gopal** MD, MS^1,6^, Nathan R. Tucker, PhD**^5,13^, Jessica L. Fetterman, PhD**^1^

^1^Evans Department of Medicine and The Whitaker Cardiovascular Institute, Boston University Chobanian & Avedisian School of Medicine.

^2^Department of Health Sciences, Programs in Human Physiology, Sargent College of Health & Rehabilitation Sciences, Boston University.

^3^Department of Medicine, Section of Computational Biomedicine, and Clinical and Translational Science Institute, Boston University Chobanian & Avedisian School of Medicine.

^4^Department of Pathology and Laboratory Medicine, Boston University Chobanian & Avedisian School of Medicine.

^5^Departments of Pharmacology and Medicine, SUNY Upstate Medical University.

^6^Section of Cardiovascular Medicine, Department of Medicine, Boston Medical Center, Boston University Chobanian & Avedisian School of Medicine.

^7^Department of Epidemiology, Boston University School of Public Health.

^8^Framingham Heart Study, Framingham MA, USA

^9^Population Sciences Branch National Heart, Lung, and Blood, National Institutes of Health, Bethesda, MD, USA.

^10^Department of Pathology, Brigham and Women’s Hospital, Harvard Medical School.

^11^University of Chicago Medicine Comprehensive Cancer Center Metabolomics Platform.

^12^Department of Biostatistics, Boston University School of Public Health.

^13^Cardiovascular Disease Initiative, Broad Institute of MIT and Harvard.

*Denotes equal contribution as 1^st^ author

**Denotes equal contribution as senior/corresponding author

**Supplemental Figure 1.** Differential Abundance of Subclasses of VSMCs in Hypertensive vs Normotensive Hearts …. Pg 3

**Figure S1:** Differential Abundance of Subclasses of VSMCs in Hypertensive vs Normotensive Hearts


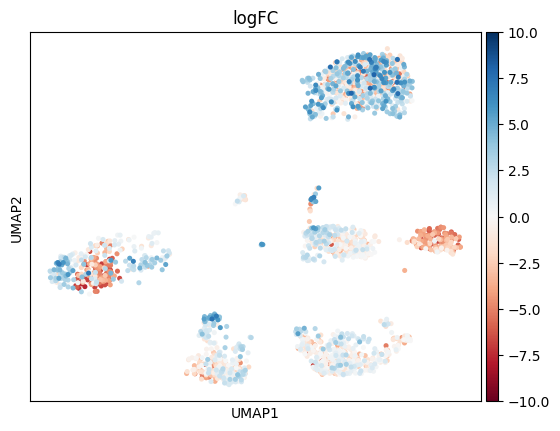

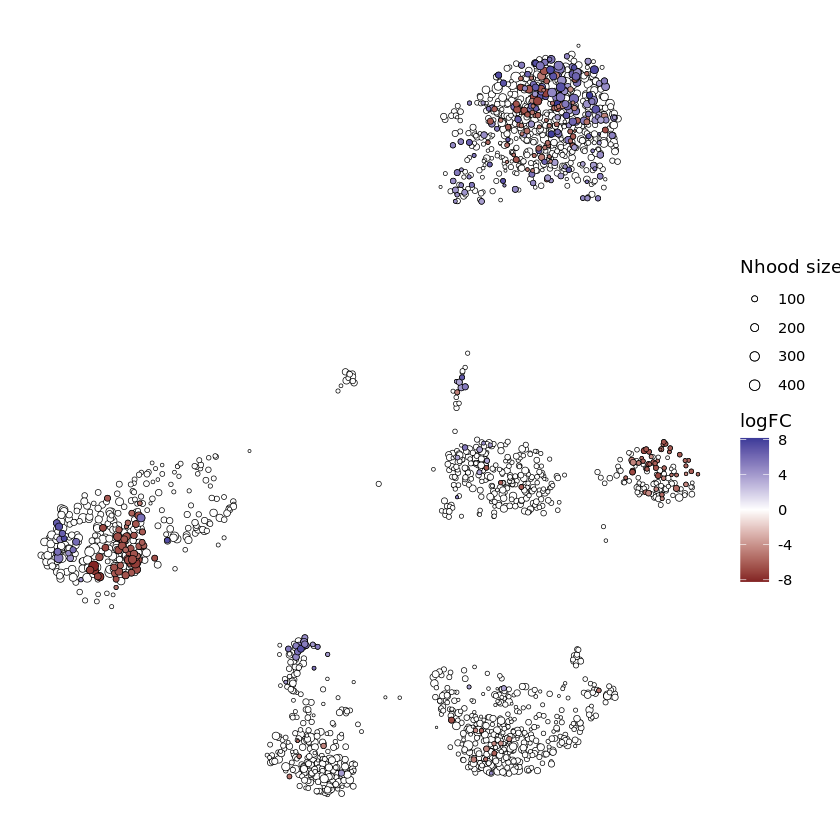


**A**

**B**

**Legend: A)** Neighborhood-level differential abundance on global UMAP embedding. Points represent neighborhoods positioned at the centroid of its member cells in UMAP space. Points are colored regardless of significance (NTN= Blue, HTN=Red, No Enrichment =White). Color intensity indicates the magnitude of the log₂ fold-change in neighborhood abundance between conditions. **B**) Neighborhood-level differential abundance on global UMAP embedding. Points represent neighborhoods positioned at the centroid of its member cells in UMAP space. Statistically significant enrichment (FDR<0.15, p-value < 0.05) is indicated by color (NTN= Blue, HTN=Red, No Enrichment = White) and was determined by the log₂ fold-change in neighborhood abundance between conditions estimated with an edgeR negative-binomial GLM; color intensity is used to represent the magnitude of the log fold change.
